# Age-Stratified Patterns of Diagnostic Delay in Pertussis

**DOI:** 10.64898/2026.09.25.26364016

**Published:** 2026-10-01

**Authors:** Emily A. Serman, Iromi Jayawardena, Carolina Liskey, Robin Lacroix, Alain H. Litwin, Jihad S. Obeid, Scott R. Curry, Lior Rennert

## Abstract

**Background:**

Pertussis has resurged in the post-COVID-19 era, yet diagnostic delay remains a barrier to timely treatment and transmission interruption, particularly among adults.

**Methods:**

Retrospective observational cohort study of 787 patients with confirmed pertussis from January 2016 through March 2026 across two South Carolina health systems. Electronic health record data from the 60 days preceding confirmation were used to characterize diagnostic timing (same-day vs. delayed), delay duration, and respiratory pathogen testing patterns including pertussis test modality.

**Results:**

Adults accounted for 48.4% of cases (median age 16 years [IQR 4–52]; 61.6% female). Same-day diagnosis was highest among children aged 1–4 years (75.4%) and lowest among adults aged 50 and older (approximately 50%). Among the 305 patients (38.8%) with delayed diagnosis, median delay ranged from 7–12 days in children to 22.5–26.5 days in adults aged 50 and older. Delay did not vary significantly by COVID-19 period, race, ethnicity, or insurance type. Testing breadth was greater in inpatient and emergency settings than outpatient. Pertussis testing was predominantly PCR-based in children but serologic in adults, potentially reflecting later clinical presentation relative to illness onset; however, the near-absence of PCR testing in older adults may contribute to missed diagnoses during the early illness window when PCR sensitivity is highest.

**Conclusions:**

Older adults experienced markedly delayed pertussis diagnoses compared with children. Earlier consideration of pertussis in adults with prolonged cough, including PCR testing early in illness before serologic conversion, may reduce diagnostic delay and facilitate timelier treatment and contact tracing.

**Summary:**

Among 787 confirmed pertussis patients across two health systems, adults aged 50 and older experienced diagnostic delays two to three times longer than children, likely driven by atypical symptom presentation and late-stage testing patterns that fall outside optimal diagnostic windows.

## Full Text Availability

The license terms selected by the author(s) for this preprint version do not permit archiving in PMC. The full text is available from the preprint server.

